# Round the Hospitals

**Published:** 1920-08-21

**Authors:** 


### ROUND THE HOSPITALS.

THE income and expenditure account of the Nightingale Fund for last year shows a prosperity which in the present state of hospital affairs is a refreshing novelty. The income from invested funds amounts to £1,613, that from paying probationers £544. The expenses of training are £1,395; sundry expenses, including books, printing, and secretarial, £175; King's College for Women, tuition fees and one term's fees for residence, £185. The balance of excess income for the year is £397, which is added to previous balances of £2,922 and has been invested. The matron records her thanks to the many old Nightingale sisters and nurses who returned in 1914 to take the place of those on war

**ROUND THE HOSPITALS—(*continued*).**

service. Had it not been for their help the continuity of the teaching in the wards would not have been possible. Miss Coode, Sister of the Preliminary Training School, and Miss Gullan, Sister Tutor, remained at their civil posts, and thus the Nightingale probationers during the war received the highest standard of theoretical and practical training. Miss Lloyd Still gives the names of twenty-two sisters who returned or remained on at civil work during the war years, and expresses herself as "proud and grateful that the Nightingale School has again risen to the claims of its hospital both for civil and military work."

---

Lord Allenby's appeal for a new modern hospital at Cairo has brought into prominence the fact that there is at present no well-equipped nurse-training centre in Egypt. The Anglo-American Hospital partakes of the nature of a nursing home, and is staffed by trained nurses, matron, theatre sister, and six others. Little has been done in Egypt in the way of experiments with native or Cairene probationers, and there are grave impediments, due principally to religious customs, to their employment. But Cairo presents a medley of races. At the Census in 1907 there were found to be over 46,000 Europeans in the city, and it is probable that among them a considerable number of women could be found willing and suitable to be trained as nurses. The need for them is greater than could possibly be supplied by inducing trained nurses from other countries to settle there. It is high time a beginning was made in the development of trained nurses in Egypt on the spot.

---

We are informed that the General Nursing Council for Scotland have, with the sanction of the Scottish Board of Health, appointed Mr. W. S. Farmer, solicitor, 13 Melville Street, Edinburgh, as Registrar of the Council. No doubt Mr. Farmer is well qualified to perform the formal duties of his new office, but we regret the appointment for two reasons. The first is that Scottish nurses would undoubtedly have preferred to find themselves in communication with one of their own sex and profession in applying for registration and entering into the minute particulars which may often be required. The second is that the new Registrar must, from the nature of his own profession, be debarred from surrendering his whole time and interest to the cause of Scottish registration.

---

There is some hope that the next Census, to be taken on the last Sunday of April, 1921, will really throw light on the vexed question of the number of trained nurses in the kingdom. Several questions have been omitted in the schedule, and among them that about "infirmities," but closer inquiries will be made into occupation, industries, and education, which if well framed should pierce through the obscurity presented by the word "nurse," and reveal the number of persons engaged in nursing the sick, as distinguished from those employed in attendance on the young. The particulars have been framed under the guidance of the Ministry of Health, in whose province it lies to gauge the number of trained nurses. It would be very useful to know the number of women who had received training as nurses, even though they may not now be following their profession.

---

The nursing-home "scandal" is always with us. The *Saturday Journal* is rightly inveighing against the inflated charges made in some of these establishments, whose proprietors are "profiteering on the afflicted." We are entirely in accord with the statement that "in a civilised community nursing homes should be licensed and regulated." It is a notorious abuse of the freedom of the subject that anyone can open a nursing home, in any premises, receive almost any number of patients, under-staff up to the limit of the staff's endurance, and palm off as nurses women who, except for their uniform and a mere smattering of experience, have nothing in common with trained nurses. At the same time, there are difficulties in the way of registering nursing homes. To introduce an unimpeachable standard of construction and prohibit the use of premises which are unsatisfactory would lead to the closing down of some 75 per cent. of the existing homes, and perhaps more. It is not a moment when structural alterations can be carried out by issuing orders. Again, under-staffing is by no means always wilful. There is a great scarcity of nurses, and many reputable homes advertise in vain for them. Granted that the public is fleeced and ill-served, it must be realised that the sick would be much worse off if they could not be received anywhere at all in their extremity. The remedy is an extension of pay hospitals. But for that the public will have to wait.

---

The M.A.B. Hospital for Venereal Diseases at Sheffield Street is now starting work, and it is hoped that it will considerably relieve the venereal ward at the Chelsea Infirmary, where up till recently the women police have been taking those young women who, finding themselves attacked by this disease, have consulted the women officers of the force. It has been agreed, after consultation with representatives of the Ministry of Health and of the London County Council with the heads of the women police, that the cases to be admitted to the Sheffield Street Hospital shall be selected and brought to the hospital by the women police and by the women of the street patrol working in and about Waterloo Station. The hospital contains fifty-two beds.

---

The superintendent nurse, three charge nurses, and eight probationers at the Lincoln Union Infirmary have resigned owing to the refusal of the Board to pay attention to their grave and repeated complaints. The superintendent nurse recounts in the *Lincoln Leader* some of the abuses which made their position untenable; they include shortage of rations allowed both to the patients and the staff,

**ROUND THE HOSPITALS**—(*continued*).

insupportable conditions for the night nurses, who are on duty seventy-two hours weekly, and are so badly housed they can get scarcely any sleep, and disregard of the conditions of training laid down for probationers. As yet the reply of the Guardians to these allegations has not come to hand. It is to be hoped that their defence will be speedily forthcoming, and convincing; for the statements of the superintendent nurse amount to a serious indictment.

---

The Kingston Guardians have not advertised for a matron to take the place of Miss Sinclair. They received two applications, and the Infirmary Committee have recommended that Miss Agnes Wood, the present inspector under the Children Act, be asked to take over the appointment of temporary matron for six months, on the same salary as was given before. At the meeting of the Board on August 3 Miss Wood was interviewed and her temporary appointment was agreed to.

---

The Cheshire County Nursing Association now has seventy nursing associations affiliated to it, and held its thirteenth annual meeting in cheerful circumstances at Chester Castle lately, with Colonel Sir George Dixon in the chair. Mrs. Paget, who was unable to be present, wrote a stimulating letter in which she urged the duty of affording rest and recreation, a comfortable chair in the garden, books, and motor drives to the nurses who served the community by constant self-sacrifice. Six candidates are being trained at Plaistow, but much scarcity prevails, and there are districts without nurses for which none can be found. Dr. Meredith Young, the County M.O.H., spoke of the need for co-ordinating the work of prevention and cure, since there is serious waste of energy and money in the overlapping of these two departments. The work must be done as team work, not as individual isolated work with friction or jealousy strangling it, and a place must be found for the V.A.D. Financially the Association is doing well, thanks to a gift of £2,300 from the British Red Cross Society.

---

At the well-organised and most successful hospital fête lately held at Winchester in aid of the Royal Hants County Hospital, a great feature was the Hospital Tent, over which were printed the words which have been adopted as a motto in the recent rally in aid of the funds: "If you will help us when you are well, we will look after you when you are sick." The Tent was in charge of the matron, Miss Carpenter Turner, and the nursing staff of the hospital, who had been largely instrumental in making and collecting the articles for sale, and it proved the most popular of all the attractions.

---

A crowded meeting of nursing officers and sisters of the St. John Ambulance Brigade has been held in Newcastle at which Miss Abraham, superintendent of the Cathedral Nursing Association, explained a scheme by which the Nursing Association could assist in the training of the St. John nurses. She described the work which the district nurses did and the difficulties they encountered in their patients' homes, and outlined a system which would give a St. John nurse a course of several months under the personal supervision of a Cathedral nurse, who would demonstrate the right way of dealing with nursing difficulties, and the easiest method of nursing under adverse conditions. The scheme has the cordial support of the Divisional Surgeon, Dr. Rutter, who laid stress on the ever-increasing shortage of nurses in the Newcastle area. Before the meeting ended several volunteers gave in their names for an immediate course and many others entered for vacancies in the future.

---

The British Red Cross Society has made a handsome gift of £150,000 to the Australian Branch in commemoration of the close co-operation that has existed between the Red Cross Societies of the various Dominions in relieving the sufferings caused by war. The sum of £25,000 has been allocated to each State to be distributed (*a*) to institutions and nursing organisations which primarily benefit sailors and soldiers and their dependants; (*b*) to purposes connected with the larger field of remedial and preventive work on which the Australian Red Cross is entering as a member of the International League of Red Cross Societies. We are particularly glad to hear that the Bush Nursing Societies of Victoria, New South Wales, and Tasmania are to have £10,000 each, while that of Queensland receives £15,000 to extend its work in country districts where soldiers are settled, and Western Australia is to have £15,000 to set up the Bush Nursing Association it lacks and sorely needs.

---

V.A.D. members who have served for two years in the wards of a properly equipped military hospital, not for convalescents, are now received for fever training in the wards of the M.A.B. institutions for eighteen months in lieu of two years, and the salaries of probationers with such previous experience are to be increased according to scale at the expiration of six months' service. The question of altering the name of the Metropolitan Asylums Board to Metropolitan Health Board is under consideration.

---

We are glad that the American Army Nurse Corps has succeeded in obtaining relative military rank. The Army nurses experienced great difficulty in the United States in receiving their proper position, and were often exposed to unnecessary hardships because no provision was made for them as "officers." We hope the new names they have acquired will have the desired effect in setting their claims to consideration in the Army as high as they ought to be. At the same time, it must be remembered that public recognition is a plant of slow growth, and depends on much besides the name bestowed. We recall with pride the testimony given by the Matron-in-Chief of the American Army Nurse Corps as to the status, fully equal to military rank, enjoyed by British Army nurses, a testimony quoted a short time back in these columns.

**ROUND THE HOSPITALS**—(*continued*).

We have received a kind and interesting letter from "G. B. T.," a Liverpool sister, a loyal member of the College of Nursing, eager for reform in many directions, and emphatically one of those who think for themselves. There is much sound sense in her approval of patients in the voluntary hospitals, both in and out, paying what they can afford, and her plaint that "the rate-paid institutions have claimed the money while the voluntary ones depended on gifts and on the service of self-sacrifice by doctors and nurses" will be endorsed by many. We are heartily in agreement with her earnest desire to see "a trained nurse of refined intelligence" installed wherever there are infants to be tended, or aged or weak-minded persons, as also to see the Ministry of Health exercising supervision over convents and "all closed-up places." In reply to the suggestion that the College should advise nurses desirous of taking up mission work, we are happy to inform our correspondent that at the Information and Enquiry Bureau, 7 Henrietta Street, Cavendish Square, Miss Herbert gives personal interviews and advice to all members who desire to consult her about such points. We hope to deal with the uses of registration on another occasion.

The Edinburgh Medical Missionary Society puts forth a moving appeal in its Quarterly Paper for help in reorganising the hospitals in Nazareth and Damascus which are under its charge, and were engulfed in the war, as well as for money and personal help in other parts of the mission-field. At the present moment it appears some of the hospitals under the charge of the mission are actually standing idle because of the lack of men and women to carry on the work, while many are seriously understaffed. Interesting particulars are given of the medical and nursing work done in the Victoria Hospital, Damascus, where Sister Mann, R.R.C., is superintendent.

There is a gratifying increase in the number of midwives added to the Scottish Roll after the recent examinations. Successful candidates number 112, of whom twenty-nine passed at Edinburgh, sixty-six at Glasgow, and seventeen at Dundee.

